# Iatrogenic Injury of Profunda Femoris Artery Branches after Intertrochanteric Hip Screw Fixation for Intertrochanteric Femoral Fracture: A Case Report and Literature Review

**DOI:** 10.1155/2014/694235

**Published:** 2014-02-05

**Authors:** Nikolaos Patelis, Andreas Koutsoumpelis, Konstantinos Papoutsis, George Kouvelos, Chrysovalantis Vergadis, Anastasios Mourikis, Sotiris E. Georgopoulos

**Affiliations:** ^1^Department of Surgery, Subdivision of Vascular Surgery, Laiko University Hospital, 17 Ag. Thoma Street, 11527 Athens, Greece; ^2^Department of Radiology, Laiko University Hospital, 17 Ag. Thoma Street, 11527 Athens, Greece; ^3^Department of Orthopedics, Laiko University Hospital, 17 Ag. Thoma Street, 11527 Athens, Greece

## Abstract

A case of arterial rupture of the profunda femoris arterial branches, following dynamic hip screw (DHS) fixation for an intertrochanteric femoral fracture, is presented. Bleeding is controlled by coil embolization, but, later on, the patient underwent orthopedic material removal due to an infection of a large femoral hematoma.

## 1. Introduction

Pseudoaneurysms and hemorrhage of the profunda femoris artery (PFA) are rare injuries and have been reported following trauma or orthopedic procedures performed in the proximal femur [1–4]. Pseudoaneurysms or hemorrhage of the PFA following dynamic hip screw fixation (DHS) for an intertrochanteric femoral fracture constitutes 0.2% of all PFA injury cases [5]. Presentation may be acute or delayed [6]. If not diagnosed properly, this injury can be life- or limb-threatening.

## 2. Case Report

A 92-years-old female was referred to the department of vascular surgery due to an enlarging hematoma of the left thigh. At the time of admission, the patient had fever (>38°C), anemia (Ht 32.9% and Hb 10.7 *μ*g/dL), but she was hemodynamically stable and in good general condition. Both lower extremities had palpable peripheral pulses.

Two months earlier, the patient underwent an intertrochanteric femur fracture repair using DHS fixation, a procedure that took place in another hospital. During the postoperative period, there has been a gradual but significant decrease in the hematocrit and hemoglobin levels to 22.5% and 7.1 *μ*g/dL, respectively, despite repeated transfusions. Ultrasound scans performed postoperatively showed a hematoma gradually increasing in diameter, from 9 cm initially to >20 cm. At the 25th postoperative day, since the enlargement of the thigh hematoma was halted and there were no signs of lower extremity ischemia, the patient was considered stable enough to be discharged.

**Figure 1:**
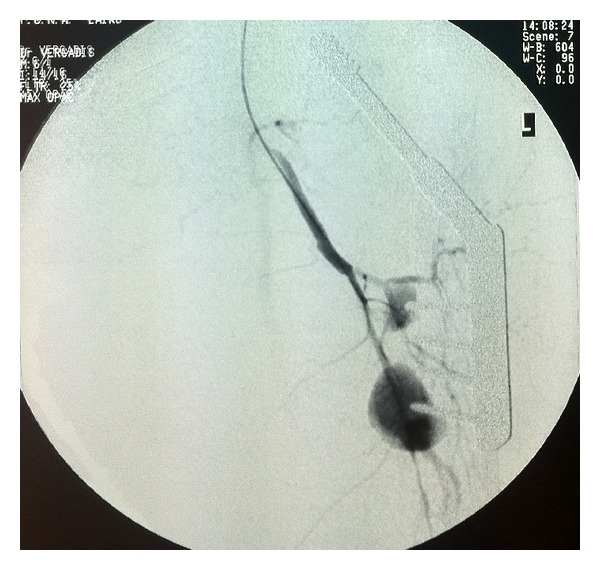
Hemorrhage fed by two perforating arteries, located at the tips of the first and fourth screws.

**Figure 2:**
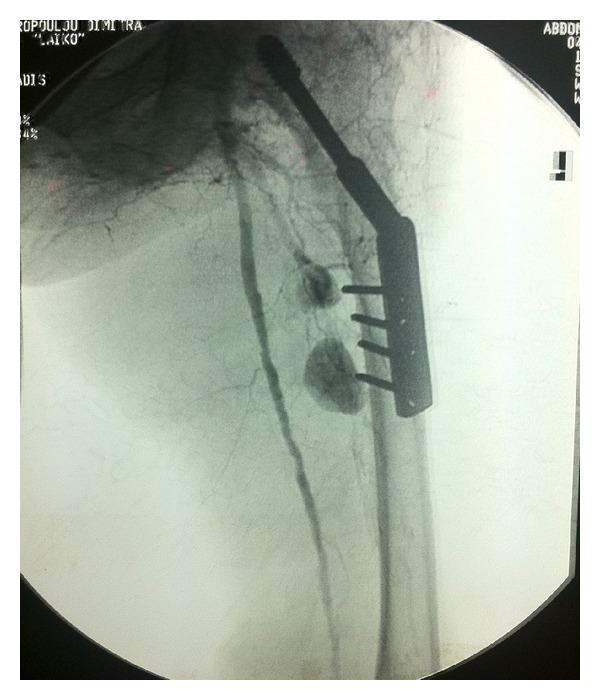
Extravasation of the contrast agent in the deep intramuscular space of the thigh. Superficial femoral artery is visibly patent.

**Figure 3:**
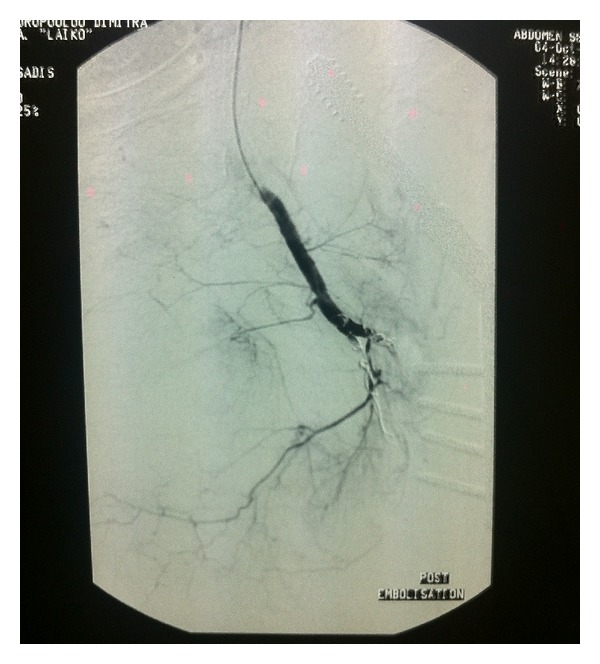
Successful coil embolisation of the two feeding arteries. No extravasation of the contrast agent is visible.

In the day following her admission to our department, an angiography was performed, that showed hemorrhage by two perforating arteries at the tip of the first and fourth orthopedic screws (Figures 1 and 2). A patent superficial femoral and popliteal artery with a patent anterior tibial artery was also demonstrated. Percutaneous transarterial embolisation with coils was performed successfully (Figure 3).

Patient's postoperative course was uneventful. At six-month follow-up, the patient was hospitalized once more due to infected orthopedic materials. She has been hemodynamically stable, without signs of bleeding from the site.

## 3. Discussion

The PFA typically gives rise to three perforating arteries that lie close to the linea aspera of the femur and therefore are vulnerable to traumatic or iatrogenic injuries related to femoral fractures and their surgical repair.

Pseudoaneurysms or hemorrhage of the PFA following femoral trauma or orthopedic repair remains rare. An English-language literature search on Medline using the terms false aneurysm, pseudoaneurysm, and pseudo-aneurysm in conjunction with the terms femoral or femur fracture returned 47 papers reporting a total of 61 cases of pseudoaneurysms following repair of proximal femoral shaft fractures since 1964 [6]. In the authors' opinion, this postoperative complication is underreported in literature; more data are necessary to support this opinion based only on experience (evidence level III).

Pseudoaneurysms or hemorrhage of the PFA may be caused by different mechanisms [7], most frequently by pressure of a sharp bone fragment, the tip of protruding cortical screws or the distal locking screw, or Gamma nail. Other causes are less frequent. Sometimes, a pseudoaneurysm or hemorrhage may become evident as late as several weeks or years after the arterial wall injury has occurred.

Acute bleeding presents with tachycardia, hypotension, rapid hematocrit decrease, rapid swelling of the thigh with palpable pulsation, and pain caused by the pressure built-up. An audible bruit might be present as well. This clinical onset is more frequent when the arterial injury is caused by fractured bone during injury or repair or overpenetration by drills, retractors, and screw tips [8].

On the contrary, as in our case, slow hemorrhage presents with a slowly growing swelling and increasing pain. Gradual erosion of the arterial wall by a protruding fixation screw tip is the most frequent cause. Atheromatic arterial wall should be considered more prone to erosion than a normal arterial wall [9]. Our patient remained hemodynamically stable for a long period of time with close to normal systolic blood pressure and insignificantly tachycardic.

Diagnosing arterial injury after DHS fixation is difficult because of its atypical clinical picture. Other postoperative complications can lead to thigh swelling, such as deep vein thrombosis, or be a consequence of the trauma that has caused the fracture. Other clinical signs, such as presence of a palpable bruit and pulsatility of the swelling, are not useful to differential diagnosis since pseudoaneurysms and hematoma after PFA injury are located deep in the intramuscular space. Signs of distal extremity ischemia are also inappropriate for differential diagnosis, as they may be absent if the arterial injury is minor.

The diagnosis can be set using duplex ultrasonography, computer tomography (CT), CT contrast angiography, conventional angiography, and magnetic resonance imaging (MRI). All these methods are able to determine the localization of the vascular lesion and the subsequent bleeding. The differential diagnosis of a femoral swelling should include deep vein thrombosis and bleeding due to soft tissue sarcomas.

The embolisation of the PFA, or its branches, is the therapy of choice in those cases where the superficial femoral artery (SFA) is patent. Otherwise, when an obstructed or injured SFA is present, reconstruction of the SFA is the available therapy.

In the last years, endovascular repair of PFA hemorrhage or pseudoaneurysms with covered stents have become feasible [10–12]. The percutaneous injection of thrombin into the pseudoaneurysm under duplex ultrasound guidance is a therapeutic option that avoids the transarterial catheterisation as in endovascular repair or the need of a sizable incision and exposure of the PFA through a hematoma as in the open repair [13]. There is limited experience with this technique and more studies are necessary.

The diagnosis of pseudoaneurysm of the femoral artery following trauma or orthopedic procedures requires awareness and a high index of suspicion. Due to the rarity of the condition, the early use of medical imaging is highly recommended when available. Otherwise, patients should be transferred to a vascular unit for further management. Although many orthopedic procedures are considered as simple procedures, previously described symptoms should arouse suspicion for a possible occurrence of pseudoaneurysm or hemorrhage.
